# Geranylgeranylacetone Ameliorates Beta-Amyloid Toxicity and Extends Lifespan *via* the Heat Shock Response in *Caenorhabditis elegans*

**DOI:** 10.3389/fragi.2022.846977

**Published:** 2022-04-27

**Authors:** Isiah Mossiah, Sabrina M. Perez, Taylor R. Stanley, Michaela K. Foley, Karen S. Kim Guisbert, Eric Guisbert

**Affiliations:** Department of Biomedical and Chemical Engineering and Sciences, Florida Institute of Technology, Melbourne, FL, United States

**Keywords:** geranylgeranylacetone, HSF1, heat shock response, Alzheimer’s disease, aging, longevity, lifespan, drug repurposing

## Abstract

Activation of a cytoprotective cellular pathway known as the heat shock response (HSR) is a promising strategy for the treatment of Alzheimer’s disease and other neurodegenerative diseases. Geranylgeranylacetone (GGA) is a commonly used anti-ulcer drug in Japan that has been shown to activate the HSR. Here, we establish *C. elegans* as a model system to investigate the effects of GGA. First, we show that GGA-mediated activation of the HSR is conserved in worms. Then, we show that GGA can ameliorate beta-amyloid toxicity in both muscle and neuronal worm Alzheimer’s disease models. Finally, we find that exposure to GGA is sufficient to extend the lifespan of wild-type worms. Significantly, the beneficial effects of GGA on both beta-amyloid toxicity and lifespan are dependent on HSR activation. Taken together, this research supports further development of GGA as a therapeutic for Alzheimer’s disease, provides evidence that HSR activation is a relevant therapeutic mechanism, and indicates that the beneficial effects of GGA are not limited to disease.

## Introduction

Translation of biomedical science discoveries into disease-modifying therapeutics is a slow and laborious process that requires enormous financial resources. The estimated cost of developing a new pharmaceutical is between 1 and 3 billion dollars (DiMasi et al., 2016; Nosengo, 2016). One analysis suggests that the returned value for investment in the pharmaceutical industry may be less than the value of the initial investments (Pushpakom et al., 2019).

Repurposing of current therapeutics is a strategy that can substantially reduce development costs (Pushpakom et al., 2019). Drug repurposing significantly reduces the attrition of candidate molecules during therapeutic development due to safety concerns, rapid metabolism, and lack of bioavailability. Therefore, drug repurposing has the potential to facilitate the development of new disease treatments. One successful example of this approach is Viagra, which was originally an antihypertensive drug that was repurposed for the treatment of erectile dysfunction.

Alzheimer’s disease (AD) is a devastating neurodegenerative disease with an urgent and desperate need for therapeutic development (Sierksma et al., 2020; Walsh and Selkoe, 2020). Two of the key pathological features of AD, senile plaques and neurofibrillary tangles, are associated with protein misfolding and aggregation. In the brains of AD patients, beta-amyloid peptides accumulate in senile plaques while Tau proteins accumulate in neurofibrillary tangles. Distinct protein aggregates also accumulate in a stunning variety of other neurodegenerative diseases (Hipp et al., 2019). While the specific connections between protein folding and pathology remain an active area of research, the consequences of protein misfolding accumulation include general loss of cellular protein folding homeostasis, synaptic loss, and neuronal death. Therefore, preventing or reversing protein misfolding is being pursued as a therapeutic strategy for AD.

The heat shock response (HSR) is a cytoprotective, cellular stress response that responds to protein misfolding induced by temperature and other stresses (Guisbert and Morimoto, 2012). The HSR is mediated by the highly conserved HSF1 transcription factor. HSF1 regulates a suite of beneficial heat shock genes, including molecular chaperones that directly facilitate the refolding and/or degradation of misfolded proteins.

Genetic activation of HSR genes has been shown to have beneficial effects in disease models for Alzheimer’s disease and other neurodegenerative diseases. For example, overexpression of the HSP70 heat shock gene suppresses toxicity in a mouse AD model (Hoshino et al., 2011). Furthermore, HSR activation has been shown to extend lifespan even in the absence of disease (Hsu et al., 2003; Morley and Morimoto, 2004). These results have prompted efforts to identify and develop small molecule HSR inducers (Calamini et al., 2011; Neef et al., 2011; West et al., 2012).

Geranylgeranylacetone (GGA), also known as Teprenone, is a drug that has had widespread use in Japan as an anti-ulcer agent since the 1980s (Zeng et al., 2018). While the mechanism of action for this drug remains incompletely characterized, it has been shown to activate the HSR in cultured cells (Hirakawa et al., 1996). HSR activation by GGA occurs via disruption of the interaction between the HSP70 moleculer chaperone and HSF1, as HSP70 is a key negative regulator of HSF1 (Otaka et al., 2007).

Administration of GGA was found to have dramatic effects in a mouse Alzheimer’s disease model. GGA treated APP23 mice exhibited decreased levels of beta-amyloid peptide, beta-amyloid aggregates, and synaptic loss (Sun et al., 2017). Importantly, these benefits are accompanied by improvements in cognitive function. Furthermore, these effects occurred during oral administration of GGA, indicating that GGA can cross the blood-brain barrier (Katsuno et al., 2005).

The promising results with GGA in the mouse Alzheimer’s disease model and its established clinical use in humans makes GGA an ideal candidate for drug repurposing. Continued development of GGA would benefit from a model system that is experimentally feasible and genetically tractable. Therefore, we establish *Caenorhabditis elegans* as a model to study the mechanism of GGA and determine if the beneficial effects of GGA against beta-amyloid toxicity requires HSR activation.

## Materials and Methods

### Nematode Strains and Cultures

Nematodes were maintained using standard laboratory techniques on NGM plates seeded with OP50 bacteria at 20°C unless otherwise indicated (Brenner, 1974). Worms were age-matched by bleaching with hypochlorite (NaOCl) and hatching overnight in M9 buffer or with synchronized egg-laying. The following strains were used: 1) N2 (wild-type) (Brenner, 1974); 2) CL 2006 [pCL12 (*unc-54*/human Aβ peptide 3-42 minigene) + *rol-6(su1006)*] (Link, 1995); 3) CL2355 [pCL45 (*snb-1*:Aβ 1-42:3′ UTR (long) + *mtl-2:GFP*]I (Wu et al., 2006); 4) CL2122 [(pPD30.38) *unc-54* (vector) + (pCL26) *mtl-2::GFP*] (Wu et al., 2006); 5) PS3551 *hsf-1(sy441)* (Hajdu-Cronin et al., 2004); 6) CF1038 *daf-16(mu86)* (Hsin and Kenyon, 1999).

### Chemicals and Reagents

GGA was obtained from Santa Cruz Biotechnology. Diacetyl was obtained from TCI America. RNAi constructs were obtained from the Ahringer RNAi library and used for feeding RNAi as previously described (Kamath and Ahringer, 2003; Ma et al., 2017). The following oligos were used: *hsp-16.2* forward (ACT​TTA​CCA​CTA​TTT​CCG​TCC​AGC), *hsp-16.2* reverse (CCT​TGA​ACC​GCT​TCT​TTC​TTT​G), *hsp-16.11* forward (GGC​TCA​GAT​GGA​ACG​TCA​A), *hsp-16.11* reverse (GCT​TGA​ACT​GCG​AGA​CAT​TG), *F44E5.5* forward (CAA​CTG​CTG​GTG​ATA​CCC​ATC​TC), *F44E5.5* reverse (CTT​GAA​AGT​GTT​CTC​TTG​GCA​CG), *hsp-70* forward (GTA​CTA​CGT​ACT​CAT​GTG​TCG​GTA​TTT​ATC), *hsp-70* reverse (ACG​GGC​TTT​CCT​TGT​TTT​CC), *cdc-42* forward (TTT​GCT​TCT​CCG​TGG​TTG​CTC​C), and *cdc-42* reverse (TCC​GTT​GAC​ACT​GGT​TTC​TGC​TTG).

### RT-qPCR

N2 worms were grown at 20°C on NGM control or 10 μM GGA plates starting at the L1 larval stage until day one of adulthood. RNA was isolated using Trizol Reagent (Thermo Fisher) as previously described with the addition of a freeze-thaw in liquid nitrogen and use of a Direct-zol RNA kit (Zymo Research) (Golden et al., 2020). RNA was reverse transcribed with an iScript cDNA Synthesis Kit (Bio-Rad) and RT-qPCR was performed using an iTaq Universal SYBR Green Supermix (Bio-Rad) and the CFX Connect cycler (Bio-Rad). HSR gene expression was normalized to expression of *cdc-42*. All qPCR experiments were performed with biological triplicates and p-values were calculated using the Student’s t-test.

### Paralysis Assay

Transgenic CL 2006 (muscle beta-amyloid) worms were grown at 20°C on NGM control or 10 μM GGA plates seeded with empty vector RNAi (L4440) or *hsf-1* RNAi containing bacteria starting at the L1 larval stage. Worms were scored for paralysis starting on day 1 of adulthood. Individuals were considered paralyzed if the body of the worm did not move when gently prodded. Worms were transferred by picking onto new plates as needed to separate adults from eggs and larva. Data was analyzed using OASIS 2 (Han et al., 2016).

### Associative Memory Assay

Transgenic CL2122 (control) and CL2355 (neuronal beta-amyloid) worms were grown at 16°C on NGM control or 10 μM GGA plates starting at the L1 larval stage until day one of adulthood. Then, worms were shifted to 25°C for 24 h to induce neuronal beta-amyloid expression. Day 2 adults were conditioned by transferring to bacteria-free NGM plates with 6 μL of 0.04% diacetyl added to the plate lid (Dosanjh et al., 2010). Control worms were transferred to bacteria-free NGM plates but not exposed to diacetyl. Plates were inverted and incubated for 2 h at 25°C. The chemotaxis assay was conducted by spotting 2 µL of 0.04% diacetyl 1 inch from the center of the assay plate on one side and 95% ethanol on the other. Worms were quickly placed at the center of each plate, followed by an additional 2 µL of odorant and 2 µL of 1 M sodium azide in each spot to paralyze the worms. Plates were then incubated at 20°C for 1 h and immediately scored. The chemotaxis index was calculated using the following formula (number of worms at the diacetyl spot—number of worms at the ethanol spot)/total number of worms.

### Lifespan

N2, PS3551 (*hsf-1* mutant), and CF1038 (*daf-16* mutant) worms were grown at 20°C on NGM control or 10 μM GGA plates starting at the L1 or L4 larval stages. Viability was scored starting on day 1 of adulthood. Individuals were considered dead if they did not have a motile response to gentle prodding and lacked pharyngeal pumping. Worms were transferred by picking onto new control or GGA plates as needed to separate adults from eggs and larva. Data was analyzed using OASIS 2 (Han et al., 2016).

## Results

**FIGURE 1:**
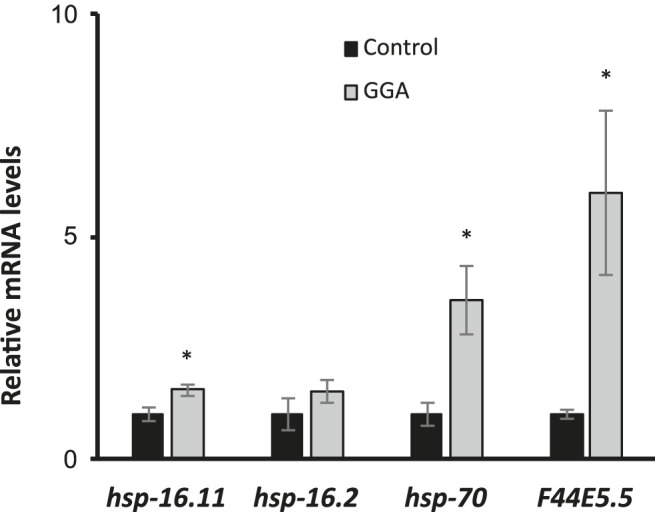
GGA activates the HSR in *C. elegans*. Wild-type (N2) worms were synchronized and incubated on NGM control or 10 μM GGA plates until day 1 of adulthood. RT-qPCR analysis showed a significant increase in basal expression of heat shock gene mRNA in worms exposed to GGA. Averages shown are from 3 biological replicates. Error bars represent SEM; * indicates p-value < 0.05 (Student’s t-test).

The ability of Geranylgeranylacetone (GGA) to ameliorate beta-amyloid toxicity in mice motivated further investigation of this drug using the genetically tractable model nematode *Caenorhabditis elegans*. Previously, GGA has been shown to activate the cytoprotective heat shock response (HSR) in cultured human cells and in mice (Zeng et al., 2018). Therefore, we first investigated the ability of GGA to activate the HSR in worms. Worms were exposed to GGA from the first larval stage (L1) to adulthood on standard NGM agar plates or NGM plates containing GGA. It was found that incubation of worms on plates containing 10 μM GGA was sufficient to increase the expression of the *hsp-16.11*, *hsp-70*, and *F44E5.5* HSR-dependent genes between 1.5-fold and 6-fold measured by RT-qPCR (Figure 1). GGA also induced a fourth HSR gene, *hsp-16.2*, 1.5-fold, but this effect was not statistically significant. The *hsp-70* and *F44E5.5* genes both encode HSP70 family proteins while *hsp-16.11* and *hsp-16.2* genes encode small HSP proteins. These results demonstrate that the effects of GGA on the HSR are conserved from humans and mice to worms and establish the basis for using *C. elegans* as model system for investigation of the effects of GGA on the HSR.

**FIGURE 2:**
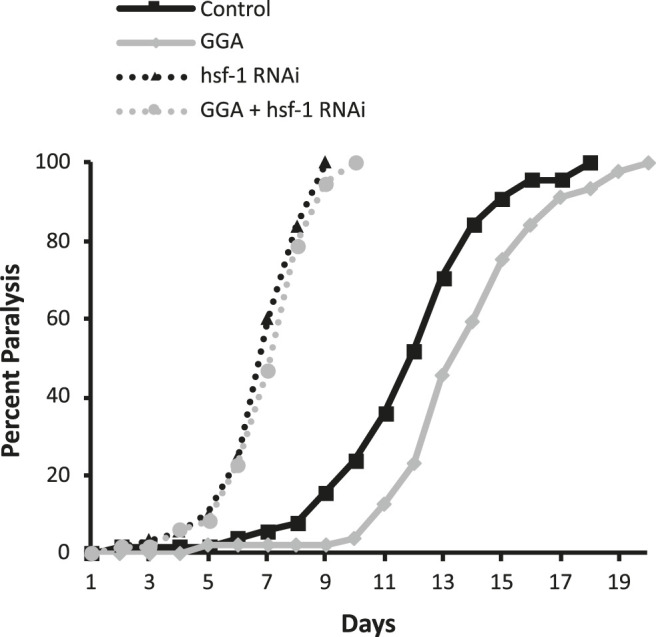
GGA alleviates paralysis in a *C. elegans* Alzheimer’s disease model. Transgenic CL2006 worms expressing beta-amyloid in muscle were synchronized and incubated on NGM control or 10 μM GGA plates seeded with empty vector (L4440) or *hsf-1* RNAi containing bacteria. Worms were scored for paralysis starting at day 1 of adulthood. Worms exposed to GGA had significantly delayed paralysis compared to control worms (p-value < 0.05, Mantel-Cox log-rank test). Each curve represents data from 3 independent trials of n ≥ 20 individuals.

Previously, GGA was shown to have beneficial effects on a mouse model for Alzheimer’s disease (AD) containing expression of the human beta-amyloid peptide (Sun et al., 2017). Therefore, we next tested whether the effects of GGA on beta-amyloid toxicity were conserved in worms. A well-established worm AD model was used that features transgenic expression of the human A-beta peptide (Link, 1995). In this model, beta-amyloid is expressed in muscle cells and the worms develop progressive paralysis as they age with 50% of the worms becoming paralyzed on day 11 of adulthood (Figure 2). We found that incubation with GGA led to a reduction in this age-dependent paralysis with the median paralysis occurring nearly 2 days later. These results indicate that GGA can ameliorate toxicity from beta-amyloid expression in a worm disease model.

Having established that the effects of GGA on HSR activation and beta-amyloid-induced paralysis were conserved in worms, we next tested whether the effects on beta-amyloid were dependent on the HSR. The HSR was inhibited by RNAi knockdown of the HSF1 transcription factor that is required for the response. Consistent with previous work, we found that inhibition of HSF1 alone enhanced the toxicity in this disease model, with median paralysis occurring around day 6 of adulthood. In contrast to its effects in wild-type worms, GGA did not have a significant effect on beta-amyloid toxicity when HSF-1 was inhibited. These results indicate that activation of the HSR is required for the beneficial effects of GGA.

**FIGURE 3:**
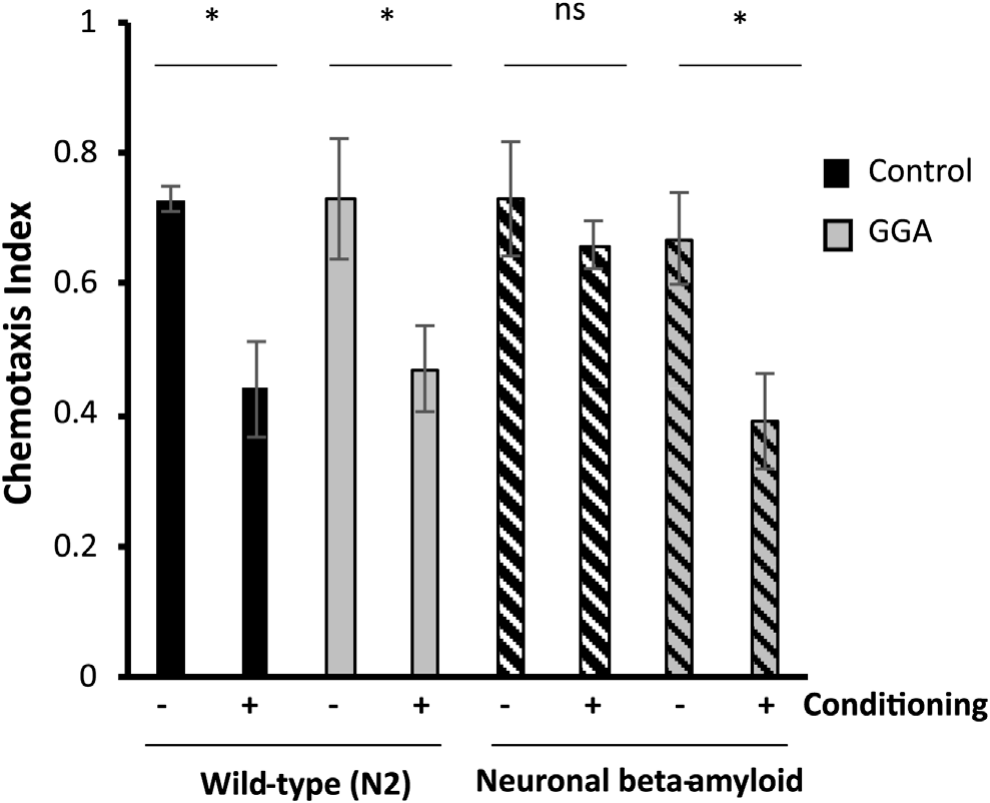
GGA restores associative learning in a neuronal Alzheimer’s disease model. Transgenic worms with or without neuronal beta-amyloid expression were synchronized and incubated on NGM control or 10 μM GGA plates at 16°C. On day one of adulthood, worms were shifted to 25°C for 24 h to induce beta-amyloid expression. Then, worms were starved in the presence or absence of the odorant diacetyl for 2 h and scored for chemotaxis. Worms expressing neuronal beta-amyloid exhibited a defect in conditioned learning that was restored upon exposure to GGA. Averages shown are from 3 biological replicates of *n* ≥ 30 adults each. Error bars represent SEM; * indicates p-value < 0.05; ns = non-significant (Student’s t-test).

Having shown that GGA can reverse the toxicity from beta-amyloid expression in muscle tissue, we next tested the effects of GGA in neurons, a more relevant tissue type for AD. In a second worm AD model, neuronal expression of human beta-amyloid has been shown to cause neuronal toxicity and results in a defect in associative learning (Dosanjh et al., 2010). In the associative learning assay, worms are simultaneously exposed to a chemoattractant and a lack of food. Upon subsequent exposures, worms display associative learning by reducing their chemotaxis towards the odorant. Naïve wild type worms exhibited chemotaxis towards diacetyl, measured by a chemotaxis index of 0.73 (Figure 3). When they were conditioned by exposure to diacetyl in the absence of food, they reduced their preference for this odorant to a chemotaxis index of 0.44. In the AD model, neuronal expression of beta-amyloid disrupted associative learning but did not affect chemotaxis of naïve worms (0.73 naïve *verses* 0.66 conditioned).

We tested the ability of GGA to reverse the associative learning defect in worms expressing neuronal beta-amyloid. GGA did not affect chemotaxis nor associative learning in wild-type worms (0.73 naïve *verses* 0.47 conditioned). Significantly, exposure to GGA restored associative learning in worms with neuronal beta-amyloid (0.68 naïve *verses* 0.39 conditioned). Taken together, these experiments demonstrate that the beneficial effects of incubation with GGA in worms extend to neurons.

**FIGURE 4:**
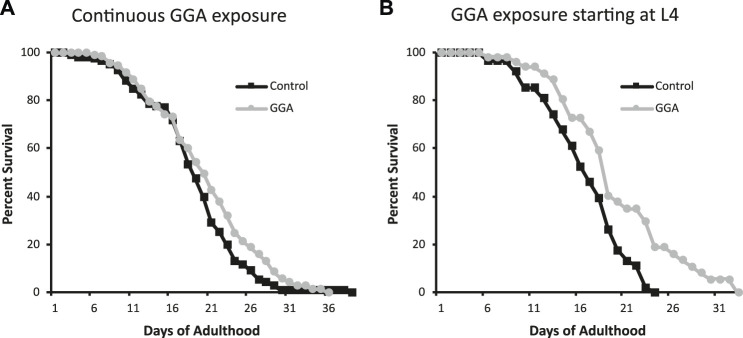
GGA extends lifespan in *C. elegans*. Wild-type (N2) worms were synchronized and incubated on NGM control plates or plates containing 10 μM GGA starting at the L1 **(A)** or L4 **(B)** larval stages. Worms were scored for viability starting at day 1 of adulthood. Worms exposed to GGA in either case lived significantly longer than control worms (p-value < 0.05, Mantel-Cox log-rank test). Each curve represents ≥3 pooled trials of *n* ≥ 20 individuals each.

**FIGURE 5:**
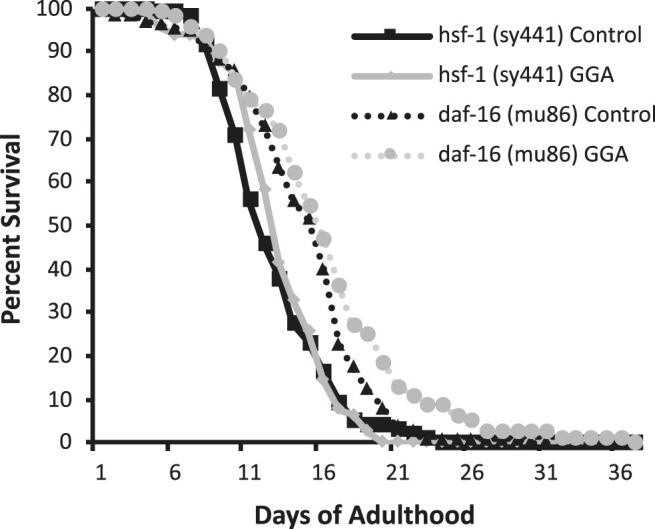
The HSR is required for GGA-mediated lifespan extension. Worms containing mutations in *daf-16 (mu86)* and *hsf-1 (sy441*) were synchronized and incubated on control NGM plates or plates containing 10 μM GGA. Worms were scored for viability starting at day 1 of adulthood. Incubation with GGA caused significant lifespan extension in *daf-16* mutant worms (p-value < 0.05 Mantel-Cox log-rank test) but not worms containing a mutation in *hsf-1*. Each curve represents ≥4 pooled trials of *n* ≥ 30 individuals each.

Having established the effects of GGA on disease models, we next tested whether its beneficial effects could extend to longevity. A wide range of protein aggregates accumulate during the normal process of aging and disruption of protein folding homeostasis, or proteostasis, is considered a hallmark of aging (David et al., 2010; López-Otín et al., 2013). Overexpression of HSF1 has been shown to extend lifespan in worms while inhibition of HSF1 causes premature aging (Hsu et al., 2003; Morley and Morimoto, 2004). Therefore, we tested whether incubation with GGA was sufficient to extend lifespan in wild-type worms. Excitingly, incubation of wild-type worms with GGA extended the mean lifespan (18.7 ± 0.5 days for control *versus* 20.2 ± 0.7 days, p-value 0.03) (Figure 4A; Table 1). This lifespan extension did not require early developmental exposure as a lifespan extension was still observed upon exposure of worms to GGA starting at the L4 stage (16.5 ± 0.7 days for control *versus* 19.9 ± 1.0 days, p-value 0.002) (Figure 4B). We tested whether this phenotype was dependent on the HSR using a worm strain containing a mutation in HSF1 that blocks HSR activation. We found that GGA was no longer was able to extend lifespan in the HSF1 mutant background (12.8 ± 0.3 days *versus* 13.1 ± 0.3 days, p-value 0.54) (Figure 5; Table 1). This dependency was specific for the HSR as the effects of GGA were not reduced in worms containing a mutation in DAF-16, a distinct longevity pathway known as insulin-like signaling (14.8 ± 0.4 *versus* 16.3 ± 0.6, p-value 0.01). Together, these data indicate that GGA can extend lifespan in worms via activation of the HSR and that its beneficial effects are not limited to neurodegenerative disease models.

**TABLE 1:** Effects of GGA on lifespan.

Strain	Condition	Replicate	Number of Worms	Mean Lifespan	Standard Error
N2	Control	Combined	180	18.72	0.48
N2	GGA	Combined	190	20.2	0.69
*daf-16(mu86)*	Control	Combined	130	14.79	0.41
*daf-16(mu86)*	GGA	Combined	140	16.3	0.55
*hsf-1(sy441)*	Control	Combined	130	12.77	0.33
*hsf-1(sy441)*	GGA	Combined	130	13.08	0.33
N2	Control (L4)	Combined	60	16.45	0.65
N2	GGA (L4)	Combined	70	19.92	0.97
N2	Control	1	30	20.05	1.19
		2	30	17.98	1.57
		3	40	19.77	1.04
		4	80	18.25	0.51
N2	GGA	1	30	21.70	1.82
		2	40	19.15	1.21
		3	40	20.13	1.07
		4	80	20.07	1.18
*daf-16(mu86)*	Control	1	30	13.77	0.78
		2	30	14.67	1.15
		3	30	16.02	0.76
		4	40	14.78	0.50
*daf-16(mu86)*	GGA	1	30	14.50	1.02
		2	40	15.92	1.03
		3	30	15.57	0.85
		4	40	18.37	1.10
*hsf-1(sy441)*	Control	1	30	12.78	0.75
		2	30	13.01	0.69
		3	30	13.34	0.76
		4	40	12.17	0.45
*hsf-1(sy441)*	GGA	1	30	13.77	0.69
		2	30	12.66	0.62
		3	30	14.26	0.69
		4	40	12.09	0.61
N2	Control (L4)	5	20	17.29	1.00
		6	20	16.05	1.28
		7	20	15.91	1.03
N2	GGA (L4)	5	35	21.12	1.16
		6	20	19.57	1.48
		7	20	18.13	1.96

## Discussion

This manuscript establishes *C. elegans* as a model system for investigation of the anti-ulcer drug geranylgeranylacetone (GGA). First, conservation of the effects of GGA in worms was shown with respect to both HSR activation and amelioration of beta-amyloid toxicity. Then, genetic manipulation of the system was used to demonstrate that activation of the HSR is required for GGA’s beneficial effects. Finally, it was discovered that GGA can also extend lifespan in wild-type worms. Taken together, this research not only supports further development of GGA as a novel Alzheimer’s disease (AD) therapeutic, but also provides a foundation that can be used for new insights into HSR regulation and lifespan.

Activation of the HSR by GGA in worms establishes a new tool in the *C. elegans* HSR field. This activation is not surprising given the high degree of conservation in the HSF1 transcription factor that mediates this response. However, worms are resistant to the effects of many small molecules due to the barrier function of their cuticle. The ability to activate the HSR independent of temperature increases and associated protein misfolding stress will enable new experimental approaches to explore HSR regulatory mechanisms. Furthermore, as HSR-inducible promoters have been one of the mainstays for ectopic expression in *C. elegans*, this tool may also be useful for other fields of biology that use worms as a model organism including developmental biology and neuroscience.

Amelioration of the detrimental effects of beta-amyloid expression in worm muscle and neuronal AD models by GGA is consistent with previous results showing that GGA has beneficial effects in a mouse AD model. However, the establishment of its effects in worms facilitated an explicit test of its dependence on the HSR. The powerful genetic tools and other advantages of this model system should set the stage for further exploration of the effects of GGA on beta-amyloid toxicity. Moreover, the use of worms can facilitate investigation of GGA in other neurodegenerative disease models such as Parkinson’s disease, ALS, and Huntington’s disease.

Excitingly, we discovered that GGA can extend the lifespan of wild-type worms. This lifespan extension is also dependent on HSF1 but not dependent on insulin-like signaling, one of the major cellular pathways that influences longevity. These results are consistent with disruption of proteostasis as a hallmark of aging, further supporting that interventions targeting this pathway can have beneficial effects. To our knowledge, our results for the first time provide experimental evidence that a small molecule HSR activator can positively affect longevity in worms. Importantly, GGA is already approved for human use to treat ulcers, indicating that it is both bioavailable and does not have substantial off-target toxicity. Therefore, these findings provide strong support for future work on GGA and other HSR inducers.

## Data Availability

The original contributions presented in the study are included in the article, further inquiries can be directed to the corresponding author.
